# Supplementary material for: STING enhances cell death through regulation of reactive oxygen species and DNA damage
Source: Nat Commun. 2021 Apr 19;12:2327. doi: 10.1038/s41467-021-22572-8 (PMC8055995; doi:10.1038/s41467-021-22572-8)
Supplement: Supplementary file 1 — Supplementary Information [file 41467_2021_22572_MOESM1_ESM.pdf]

STING Enhances Cell Death Through Regulation of Reactive Oxygen Species and DNA Damage

Supplementary Information:

Supplementary Methods

RNA Sequencing

Cells were treated as described, collected and RNA isolated using RNeasy Mini kit (Qiagen). Total RNA quality is determined by estimating the A260/A280 and A260/A230 ratios by nanodrop. RNA integrity is determined by running an Agilent Bioanalyzer gel, which measures the ratio of the ribosomal peaks. mRNA is purified from approximately 200ng of total RNA with oligo-dT beads and sheared by incubation at 94^0C in the presence of Mg (Kapa mRNA Hyper Prep). Following first-strand synthesis with random primers, second strand synthesis and A-tailing are performed with dUTP for generating strand-specific sequencing libraries. Adapter ligation with 3' dTMP overhangs are ligated to library insert fragments. Library amplification amplifies fragments carrying the appropriate adapter sequences at both ends. Strands marked with dUTP are not amplified. Indexed libraries that meet appropriate cut-offs for both are quantified by qRT-PCR using KAPA Biosystems kit and the insert size distribution was determined with the LabChip GX or Agilent Bioanalyzer. Samples were loaded onto an Illumina NovaSeq flow cell (Illumina, San Diego, CA) at a concentration that yields 25 million passing filter clusters per sample and sequenced using 100 bp paired-end sequencing according to Illumina protocols. A positive control (prepared bacteriophage Phi X library) provided by Illumina was spiked into every lane at a concentration of 0.3% to monitor sequencing quality in real time. Signal intensities were converted to individual base calls during a run using the system's Real Time Analysis (RTA) software. Primary analysis, sample de-multiplexing, and alignment to the human genome were performed

using Illumina's CASAVA 1.8.2 software suite. Reads were trimmed for quality using custom scripts, with a minimum accepted length of 45 bases. The trimmed reads were aligned to the mm10 reference genome using gencode annotation using HISAT2 for alignment and StringTie for transcript abundance estimation. The generated counts were processed with DESeq2 in R to determine statistically significantly expressed/changed genes [1]. DESEq2 uses Wald's test for each gene to determine if the log fold change is statistically significant with multiple hypothesis testing correction applied. For this current analysis were used an adjusted p-value ≤ 0.05 (effectively accepting 5% false discovery rate). GSEA Preranked analysis was completed by inputting a preranked genelist with the hallmark (v7.0) gene set database using 1000 permutations. RNA-sequencing experiments were performed in biological triplicate.

TMA Antibodies and Immunohistochemistry (IHC)/Immunofluorescence (IF)

One rabbit monoclonal STING antibody, clone D2P2F (cat # 13647S, Cell Signaling Technology, Danvers, MA) was purchased and used for automated QIF analysis using the AQUA method on the HNSCC cohort. Prior to staining this antibody on YTMA329, extensive antibody validation procedures were completed to ensure high specificity, sensitivity, and reproducibility of the antibody binding to its antigen on STING for IHC/IF assays. Our standards for validation of D2P2F included observation of expected cellular localization of STING with IF, assay reproducibility assessments, lack of STING expression in the STING-KO FaDu cells, a correlation between orthogonal methods using vendor-provided western blot evidence of D2P2F STING detection in the cell lines KARPAS and THP-1 with QIF scores of a cell line index TMA (YTMA405), and regressions with QIF analysis between D2P2F and a monoclonal mouse STING antibody, clone 723505 purchased from a second vendor (cat # MAB7169, R&D Systems,

Minneapolis, MN), that binds to an independent epitope on STING. Once these standards were met, we considered D2P2F validated for use on YTMA329 (Suppl. Figure 3).

Fresh TMA cuts were subjected to deparaffinization at 60 °C for at least 45 minutes, then incubated twice in xylene for 20 minutes. Rehydration was completed using ethanol. Antigen retrieval was performed using citrate buffer (pH = 6.0) at 97 °C for 20 minutes using a pressure boiling container (PT Module, Lab Vision). Next, slides were incubated for 30 minutes at room temperature with 0.3% hydrogen peroxide in methanol and for 30 minutes at room temperature with 0.3% bovine serum albumin with 0.05% Tween-20 blocking solution. Slides were then incubated overnight at 4 °C with a mixture of primary anti-STING antibody (clone D2P2F) at the optimal concentration of 0.5 ug/mL and a mouse monoclonal anti-human cytokeratin antibody at a 1:100 dilution (clone AE1/AE3, cat # M3515; Agilent Technologies, Santa Clara, CA, USA). The sections were incubated for 1 hour at room temperature with Alexa 546-conjugated goat anti-mouse secondary antibody (A11003, Thermo Fisher Scientific, Waltham, MA, USA) diluted 1:100 in rabbit EnVision amplification reagent (K4003, Agilent Technologies). The slides were then incubated for 10 minutes at room temperature with Cyanine 5 (Cy5) directly conjugated to tyramide at a 1:50 dilution (FP1117, Perkin-Elmer, Waltham, MA, USA). Finally, the slides were incubated for 20 minutes with 4,6-diamidino-2-phenylindole (DAPI) at a 1:500 dilution and mounted with ProLong mounting medium (ProLong Gold, P36930; Thermo Fisher Scientific).

Automated Quantitative Immunofluorescence (QIF), Statistics and Cut-Points

Automated QIF analysis with the AQUA platform allows for objective quantification of protein concentrations within different compartments and has been previously described[2,3]. The STING QIF score in the tumor compartment was calculated by dividing the sum of the STING

compartment pixel intensities by the area of cytokeratin positivity resulting in a continuous score directly proportional to the concentration of STING. Similarly, QIF scores for STING in the stromal compartment were calculated by dividing the sum of the STING compartment pixel intensities by the area of the DAPI compartment minus the tumor compartment. QIF scores for both the tumor and stromal compartments were subsequently normalized for exposure time and bit depth to be comparable for analysis. Each histospot was reviewed, and spots with staining artifacts were excluded from analysis. Log-rank tests were used to compare the PFS functions of patients with either high or low STING expression in the tumor and stromal compartments using GraphPad Prism. We split the tumors into high and low STING expression groups using the medians of the continuous QIF scores for STING in each compartment as the cut-points.

Supplementary References:

1. Love, M.I., Huber, W. & Anders, S. Moderated estimation of fold change and dispersion for RNA-seq data with DESeq2. *Genome Biol* **15**, 550 (2014).
2. Camp, R.L., Chung, G.G. & Rimm, D.L. Automated subcellular localization and quantification of protein expression in tissue microarrays. *Nat Med* **8**, 1323-1327 (2002).
3. McCabe, A., Dolled-Filhart, M., Camp, R.L. & Rimm, D.L. Automated quantitative analysis (AQUA) of in situ protein expression, antibody concentration, and prognosis. *J Natl Cancer Inst* **97**, 1808-1815 (2005).

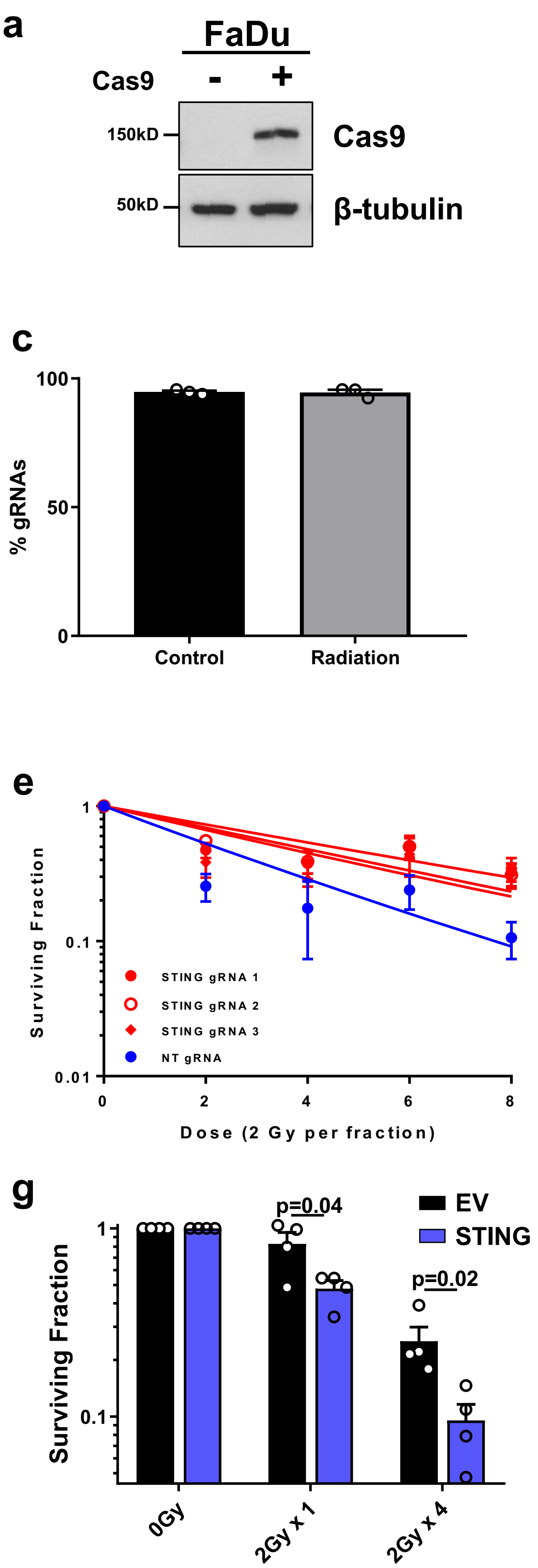

Supplementary
Figure 1

Supplementary Figure 1. **STING Loss Enhances Resistance to DNA-Damaging Therapies. a.** Immunoblots of FaDu cells showing successful expression of Cas9 as compared to parental cell line and are representative of 2 independent experiments. **b.** Clonogenic survival analysis of parental (WT) and Cas9 expressing FaDu cells (error bars represent SEM of n = 3 independent experiments). **c.** Percentage of gRNAs present in unirradiated (control) and irradiated (radiation) screening replicates. Data represent mean +/- SEM for n=3 independent screening replicates. Clonogenic survival curves in FaDu (**d**) and Detroit562 (**e**) with multiple STING targeting gRNAs showing increased radioresistance with STING loss in clonogenic survival assays. In **d-e** error bars represent SEM from 3 independent experiments. **f.** Immunoblot showing re-expression of STING in FaDu STING KO cells as compared with KO or WT cells and are representative of 2 technical replicates. **g.** Quantification of clonogenic survival analysis of FaDu STING KO cells with EV or STING re-expression. Error bars represent SEM from 4 independent experiments. *P -values from unpaired, two-tailed t-test without multiple comparison correction.

Supplementary Figure 2

a

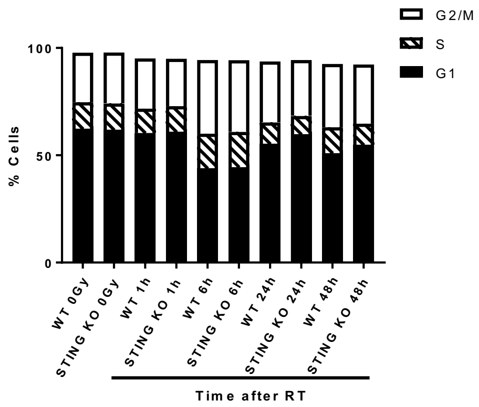

Supplementary Figure 2. STING loss does not alter cell cycle distribution at baseline or after radiation. a. Cell cycle distribution of FaDu WT or STING knockout (KO) cells at baseline (0Gy) or at the indicated times after RT (2Gy x 4). Results represent the average from 3 independent experiments.

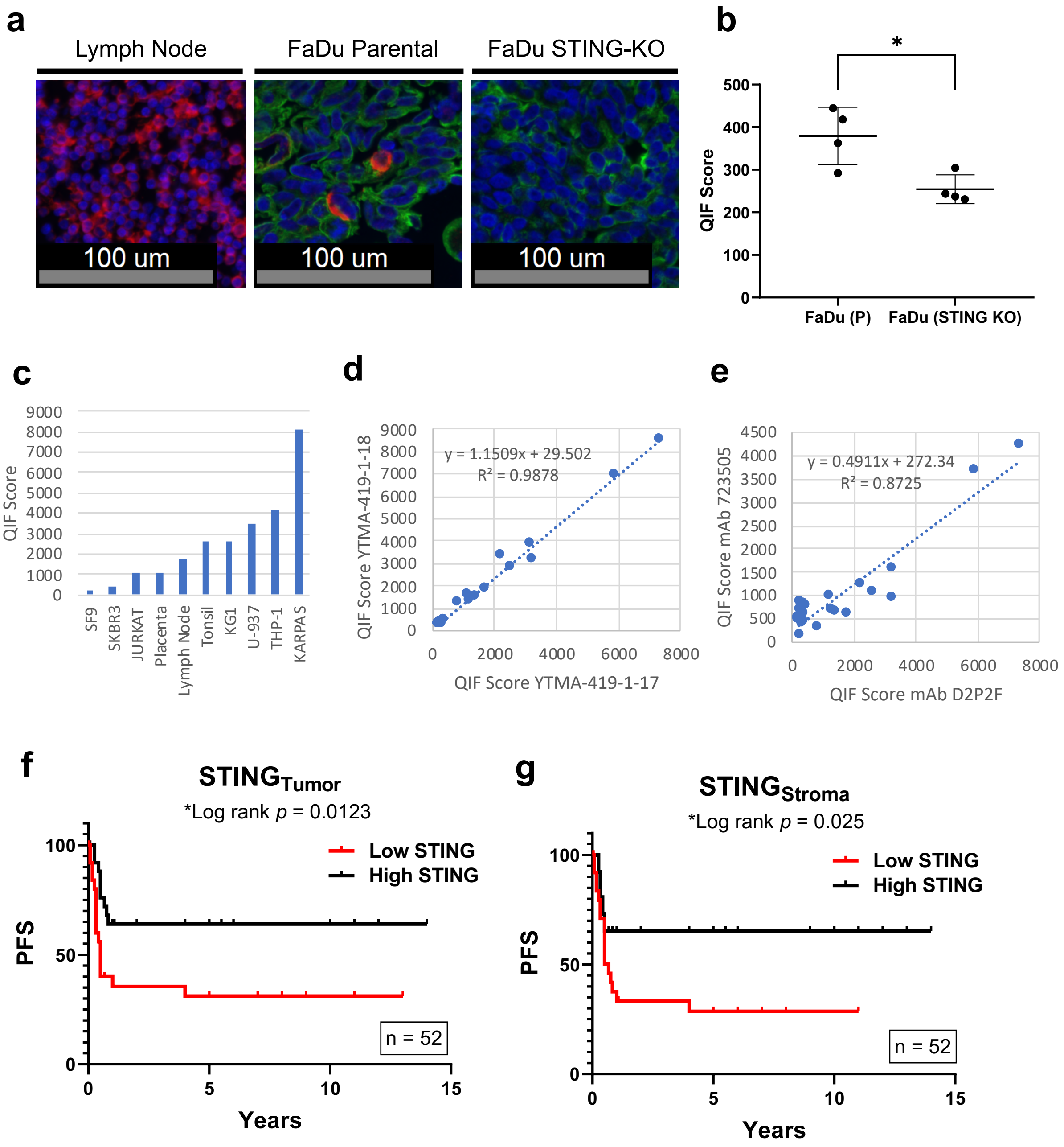

Supplementary Figure 3

Supplementary Figure 3. Antibody Validation of STING antibody, clone D2P2F for IHF/IF Assays and TMA Analysis. a. Representative STING expression in lymph node tissue, parental FaDu and FaDu STING KO cells. STING protein is represented in red, cytokeratin (tumor) in green, and DAPI (nuclei) in blue. Images are representative two independent TMA spots. **b.** Comparison of QIF scores between the parental and STING KO FaDu cell lines. Error bars represent the standard deviation of 4 biological replicates. *P= 0.016 from unpaired Student's t-test. **c.** QIF scores of the cell lines and different tissue types of YTMA405. **d.** Regression between QIF scores for two stains with D2P2F of serial sections of YTMA419 performed on different days. **e.** Regression between QIF scores for two stains of serial sections of YTMA419, utilizing two independent antibodies (D2P2F and the mouse monoclonal STING antibody, clone 723505). **f.** Kaplan-Meier curves of patients stratified by tumor STING expression (low vs high). **g.** Kaplan-Meier curves of patients stratified by stromal STING expression (low vs high). Statistical analysis in **f-g** was performed by log-rank testing with P values as indicated in graphs

a

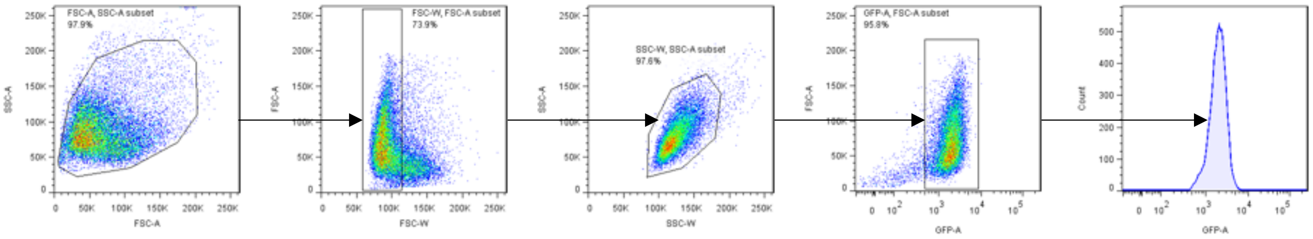

b

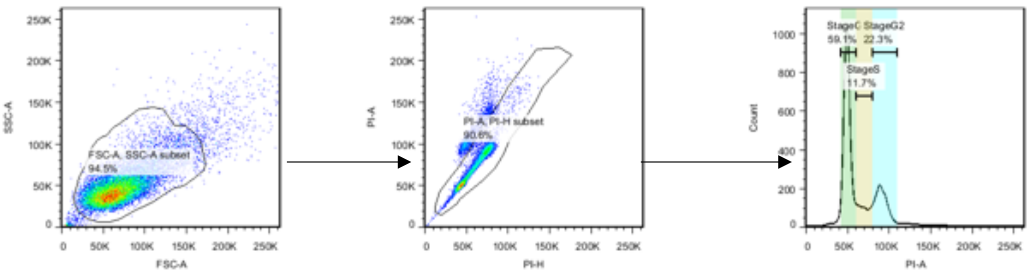

Supplementary Figure 4

Supplementary Figure 4. Flow Cytometry Gating Strategy. a. Gating strategy for CM-H2DCFDA ROS experiments. **b.** Gating strategy for cell cycle analysis.

Supplementary Table 1. Top 50 genes associated with enriched gRNAs after radiation.

Gene	P-value	FDR-Corrected P-value	Fold Change
TMEM173	2.37E-07	0.00495	2.229468784
LMCD1	4.62E-05	0.375248	1.670766359
FCGR2B	5.43E-05	0.375248	0.706357281
AQP6	7.79E-05	0.375248	1.813265272
ZNF480	8.98E-05	0.375248	1.313603613
P2RY8	0.000231	0.437894	1.575598556
TDRD12	0.000144	0.398515	1.360144566
RGS16	0.000172	0.398515	1.796688121
USP51	0.00019	0.398515	1.575380147
SAMD15	0.000191	0.398515	1.992431327
ALAD	0.000264	0.451257	1.830274454
hsa-mir-4646	0.000172	0.398515	0.879557622
KIAA0753	0.000281	0.451257	1.609642162
SLC19A2	0.000329	0.490453	1.455222748
TBC1D24	0.000429	0.59769	1.903520359
NOL7	0.000512	0.668007	1.046995325
ODAM	0.000633	0.763614	2.136426967
PINK1	0.000687	0.763614	0.826232663
TPRKB	0.000698	0.763614	1.601351481
BIN2	0.000731	0.763614	1.480392775
SCAMP5	0.000809	0.801305	1.770074399
TCF7L2	0.000844	0.801305	1.932488729
PTK6	0.000949	0.827351	1.266730833
ART3	0.00095	0.827351	1.610066191
OR2T1	0.001097	0.829348	1.460527983
TOB2	0.00116	0.829348	1.259080041
ZNF469	0.001189	0.829348	1.634455388
IL13RA2	0.001202	0.829348	1.933038002
CXorf23	0.001348	0.829348	1.792061315
ATXN10	0.001406	0.829348	1.581781142
BFSP1	0.001453	0.829348	1.196300643
HEXIM1	0.001461	0.829348	0.758941189
hsa-mir-3681	0.001038	0.829348	1.922468554
UBXN2B	0.001557	0.829348	1.681932724
TPR	0.001714	0.829348	1.01753682
DNAJB4	0.0018	0.829348	1.525783218
CLDN10	0.001812	0.829348	1.519966998
PODXL2	0.001828	0.829348	1.432157341
OPN5	0.001871	0.829348	1.577915556
CPPED1	0.001898	0.829348	1.429529112
DPY30	0.001904	0.829348	1.907006816
CYB5RL	0.001922	0.829348	1.304258649
OR52M1	0.001931	0.829348	1.48746941
ZBTB1	0.001977	0.829348	0.845572287
TRMT10B	0.002005	0.829348	1.660111336
CCBL2	0.002015	0.829348	1.616361619
hsa-mir-627	0.001423	0.829348	0.981943554
AHCTF1	0.002052	0.829348	1.64553875
NR1I3	0.002081	0.829348	1.225465611
DCAF4L1	0.002081	0.829348	1.586304796

Supplementary Table 2. Clinical Characteristics for Oropharyngeal Carcinoma Tissue Microarray Patients.

N = 52					
Sex			n	(%)	
	Male			34	65.4
	Female			18	34.6
Race			n	(%)	
	White			44	84.6
	Non-White			5	9.6
	Unknown			3	5.8
Age			n	(%)	
	Less Than 65			37	71.2
	65 and Above			15	28.8
Tobacco History			n	(%)	
	Current			15	28.8
	Former			29	53.8
	Never			7	13.5
	Unknown			1	1.9
Alcohol History			n	(%)	
	Current			23	44.2
		Abuse		6	11.5
	Former			18	34.6
		Abuse		12	23.1
	None			7	13.5
	Unknown			4	7.7
HPV Status			n	(%)	
	Positive			16	30.8
	Negative			22	42.3
	Unknown			14	26.9
Cancer Stage			n	(%)	
	I			3	5.8
	II			10	19.2
	III			5	9.6
	IV			32	61.5
		IVA		27	51.9
		IVB		5	9.6
	Unknown			2	3.8

Supplementary Table 3. Table of Antibodies Used

Antibody	Vendor	Catalog Number	RRID	Dilution
Cas9	Cell Signaling	97982	AB_2800295	1:1000
Cytokeratin	Agilent	M3515	AB_2132885	1:100 (IHC)
ISG15	Santa Cruz	sc-166755	AB_2126308	1:1000
phospho-TBK1 (s172)	Cell Signaling	5483	AB_10693472	1:1000
STING	Cell Signaling	13647	AB_2732796	1:1000 (Western Blotting) 0.5 ug/mL (IHC)
STING	R&D Systems	MAB7169	AB_10971940	0.05 ug/mL
TBK1	Cell Signaling	3504	AB_2255663	1:1000
α-Tubulin	Sigma Aldrich	T5618	AB_477579	1:1000
β-Tubulin	Cell Signaling	2128	AB_823664	1:1000
γH2AX	Millipore	05-636	AB_309864	1:500
Vinculin	Cell Signaling	13901	AB_2728768	1:1000

Supplementary Table 4. gRNA and Primer Sequences – Please see attached excel file.

Name	Sequence
**Cas9 System**	
Non-targeting gRNA 1F	CACCGCTGAAAAAGGAAGGAGTTGA
Non-targeting gRNA 1R	AAACTCAACTCCTTCCTTTTTCAGC
Non-targeting gRNA 2F	CACCGAAGATGAAAGGAAAGGCGTT
Non-targeting gRNA 2R	AAACAACGCCTTTCCTTTCATCTTC
Sting gRNA 1F	CACCGGCGGGCCGACCGCATTTGGG
Sting gRNA 1R	AAACCCCAAATGCGGTCGGCCCGCC
Sting gRNA 2F	CACCGCATATTACATCGGATATCTG
Sting gRNA 2R	AAACCAGATATCCGATGTAATATGC
Sting gRNA 3F	CACCGGGATGTTCAGTGCCTGCGAG
Sting gRNA 3R	AAACCTCGCAGGCACTGAACATCCC
**dCas9 System**	
STING dcas 1F	TTGGAGAGCAGCCAGTGTCCGGGGTTTAAGAGC
STING dcas 1R	TTAGCTCTTAAACCCCGGACACTGGCTGCTCTCCAACAAG
STING dcas 2F	TTGGGCTGCTCTGGATGATGACGGTTTAAGAGC
STING dcas 2R	TTAGCTCTTAAACCGTCATCATCCAGAGCAGCCCAACAAG
STING dcas 3F	TTGGACTCAGGCCCAGCTCATCAGTTTAAGAGC
STING dcas 3R	TTAGCTCTTAAACTGATGAGCTGGGCCTGAGTCCAACAAG
Non-targeting gRNA 1F	TTGGCTGCATGGGGCGCGAATCAGTTTAAGAGC
Non-targeting gRNA 1R	TTAGCTCTTAAACTGATTCGCGCCCCATGCAGCCAACAAG
**gRNA Amplification**	
gRNA F	AATGGACTATCATATGCTTACCGTAACTTGAAAGTATTTCG
gRNA R	CTTTAGTTTGTATGTCTGTTGCTATTATGTCTACTATTCTTTCC
**Barcodes**	
HK118	AATGATACGGCGACCACCGAGATCTACACTCTTTCCCTACACGACGCTCTTCCGATCTGATCGATAACGCATTTCTTGTGGAAAGGACGAAACACCG
HK119	AATGATACGGCGACCACCGAGATCTACACTCTTTCCCTACACGACGCTCTTCCGATCTCGATCGATACAGGTATTCTTGTGGAAAGGACGAAACACCG
HK120	AATGATACGGCGACCACCGAGATCTACACTCTTTCCCTACACGACGCTCTTCCGATCTACGATCGATAGGTAAGGTCTTGTGGAAAGGACGAAACACCG
HK121	AATGATACGGCGACCACCGAGATCTACACTCTTTCCCTACACGACGCTCTTCCGATCTTAACAATGGTCTTGTGGAAAGGACGAAACACCG
HK122	AATGATACGGCGACCACCGAGATCTACACTCTTTCCCTACACGACGCTCTTCCGATCTATACTGTATCTCTTGTGGAAAGGACGAAACACCG
HK123	AATGATACGGCGACCACCGAGATCTACACTCTTTCCCTACACGACGCTCTTCCGATCTGATAGGTCGCATCTTGTGGAAAGGACGAAACACCG
HK092	CAAGCAGAAGACGGCATACGAGATAACGCATTGTGACTGGAGTTCAGACGTGTGCTCTTCCGATCTATTCTACTATTCTTTCCCCTGCACTGT
HK093	CAAGCAGAAGACGGCATACGAGATACAGGTATGTGACTGGAGTTCAGACGTGTGCTCTTCCGATCTGATTCTACTATTCTTTCCCCTGCACTGT
HK094	CAAGCAGAAGACGGCATACGAGATAGGTAAGGGTGACTGGAGTTCAGACGTGTGCTCTTCCGATCTCGATTCTACTATTCTTTCCCCTGCACTGT
HK095	CAAGCAGAAGACGGCATACGAGATAACAATGGGTGACTGGAGTTCAGACGTGTGCTCTTCCGATCTCGATCTCTACTATTCTTTCCCCTGCACTGT
HK096	CAAGCAGAAGACGGCATACGAGATACTGTATCGTGACTGGAGTTCAGACGTGTGCTCTTCCGATCTTTCTACTATTCTTTCCCCTGCACTGT
HK097	CAAGCAGAAGACGGCATACGAGATAGGTCGCAGTGACTGGAGTTCAGACGTGTGCTCTTCCGATCTATTCTACTATTCTTTCCCCTGCACTGT
**STING Re-expression**	
BamHI-F	TAAGCAGGATCCCAGCATGCCCCACTCCAG
NotI-R	TGGCATGCGGCCGCTCAAGAGAAATCCGTGCGG
