# Supplementary material for: STING enhances cell death through regulation of reactive oxygen species and DNA damage
Source: Nat Commun. 2021 Apr 19;12:2327. doi: 10.1038/s41467-021-22572-8 (PMC8055995; doi:10.1038/s41467-021-22572-8)
Supplement: Supplementary file 3 — Description of Additional Supplementary Files [file 41467_2021_22572_MOESM3_ESM.pdf]

Description of Additional Supplementary Files

File Name: Supplementary Data 1
Description: **List of Significantly Altered Genes after STING Knockout.**

File Name: Supplementary Data 2
Description: **Gene List and Associated Fold-changes for Genes in Figure 4b.**
