# Supplementary material for: STING enhances cell death through regulation of reactive oxygen species and DNA damage
Source: Nat Commun. 2021 Apr 19;12:2327. doi: 10.1038/s41467-021-22572-8 (PMC8055995; doi:10.1038/s41467-021-22572-8)
Supplement: Supplementary file 6 — Reporting Summary [file 41467_2021_22572_MOESM6_ESM.pdf]

nature research

Corresponding author(s): *Double-blind peer review submissions: write DBPR and your manuscript number here instead of author names.*

Last updated by author(s): *YYYY-MM-DD*

Reporting Summary

Nature Research wishes to improve the reproducibility of the work that we publish. This form provides structure for consistency and transparency in reporting. For further information on Nature Research policies, see our Editorial Policies and the Editorial Policy Checklist.

Statistics

For all statistical analyses, confirm that the following items are present in the figure legend, table legend, main text, or Methods section.

n/a	Confirmed	
☐	☒	The exact sample size (*n*) for each experimental group/condition, given as a discrete number and unit of measurement
☐	☒	A statement on whether measurements were taken from distinct samples or whether the same sample was measured repeatedly
☐	☒	The statistical test(s) used AND whether they are one- or two-sided<br>*Only common tests should be described solely by name; describe more complex techniques in the Methods section.*
☒	☐	A description of all covariates tested
☐	☒	A description of any assumptions or corrections, such as tests of normality and adjustment for multiple comparisons
☐	☒	A full description of the statistical parameters including central tendency (e.g. means) or other basic estimates (e.g. regression coefficient) AND variation (e.g. standard deviation) or associated estimates of uncertainty (e.g. confidence intervals)
☐	☒	For null hypothesis testing, the test statistic (e.g. *F*, *t*, *r*) with confidence intervals, effect sizes, degrees of freedom and *P* value noted<br>*Give P values as exact values whenever suitable.*
☒	☐	For Bayesian analysis, information on the choice of priors and Markov chain Monte Carlo settings
☒	☐	For hierarchical and complex designs, identification of the appropriate level for tests and full reporting of outcomes
☒	☐	Estimates of effect sizes (e.g. Cohen's *d*, Pearson's *r*), indicating how they were calculated

Our web collection on statistics for biologists contains articles on many of the points above.

Software and code

Policy information about availability of computer code

Data collection	All software is commercially or freely available. Statistical analysis was done in Prism 7 or 8 or with MAGeCK (CRISPR Screen) or DeSeq2 as indicated in the manuscript. All tests are two-tailed unless otherwise with test indicated in manuscript.
Data analysis	All software is commercially or freely available. Results are expressed as mean ± SEM unless otherwise indicated. GraphPad Prism 7 or 8 software (GraphPad software, Inc., La Jolla, CA) was used for statistical analysis as described within Results. P-value ≤ 0.05 was considered statistically significant. CRISPR-Cas9 screen was analyzed using Cutadapt (version 1.10), Bowtie (version 0.12.8) and MAGeCK (version 0.5.6) algorithm (Ref 24; freely available). RNA-Seq data analyzed using R (version R 3.6), HISAT2 (version 2.1.0), StringTie (version 1.3.4) and the available DESeq2 algorithm (version 1.3) as described in manuscript and Ref 69. FlowJo version 7.6 was used for flow cytometry analysis. GSEA version 4.0.0 was used. OpenComet version 1.3.1 was used for comet assay.

For manuscripts utilizing custom algorithms or software that are central to the research but not yet described in published literature, software must be made available to editors and reviewers. We strongly encourage code deposition in a community repository (e.g. GitHub). See the Nature Research guidelines for submitting code & software for further information.

Data

Policy information about availability of data

All manuscripts must include a data availability statement. This statement should provide the following information, where applicable:

- Accession codes, unique identifiers, or web links for publicly available datasets
- A list of figures that have associated raw data
- A description of any restrictions on data availability

SB11285 was obtained through a research agreement with Spring Bank Pharmaceuticals (now F-Star Therapeutics). Data and materials associated with this study are available by reasonable request to the corresponding author. CRISPR screening data (GSE147084) and RNA-sequencing data (GSE147085) to the Gene

Expression Omnibus (GEO). Source data for all figures including uncropped immunoblots are available in Source Data supplemental file provided with this manuscript.

Field-specific reporting

Please select the one below that is the best fit for your research. If you are not sure, read the appropriate sections before making your selection.

☒ Life sciences ☐ Behavioural & social sciences ☐ Ecological, evolutionary & environmental sciences

For a reference copy of the document with all sections, see nature.com/documents/nr-reporting-summary-flat.pdf

Life sciences study design

All studies must disclose on these points even when the disclosure is negative.

Sample size	No statistical methods were used to determine sample size. Sample sizes are indicated in the manuscript. Sample size was determined based upon previous publications (PMIDs: 24198241, 1387891, and 30026325).
Data exclusions	No data were excluded.
Replication	Replication was performed as indicated in the methods and figure legends. In vitro experiments were repeated at least twice with similar results unless otherwise indicated in the figure legends. In vivo experiments were performed with the indicated number of mice per treatment arm.
Randomization	Mice were randomized based upon tumor size into the respective groups as indicated in the manuscript. Randomization of the patient cohort was not performed as this was a retrospective analysis of patient specimens. Randomization is not applicable for in vitro experiments as genetic knockouts or wild type cells with different treatments cannot be randomized.
Blinding	Investigators were not blinded during experiments, particularly as animal experiments required irradiation and treatment groups needed to be clear to perform the appropriate treatments. Experiments were designed with the indicated controls, and samples for comparison were collected and analyzed under the same conditions.

Reporting for specific materials, systems and methods

We require information from authors about some types of materials, experimental systems and methods used in many studies. Here, indicate whether each material, system or method listed is relevant to your study. If you are not sure if a list item applies to your research, read the appropriate section before selecting a response.

Materials & experimental systems

n/a	Involved in the study	
☐	☒	Antibodies
☐	☒	Eukaryotic cell lines
☒	☐	Palaeontology and archaeology
☐	☒	Animals and other organisms
☐	☒	Human research participants
☐	☒	Clinical data
☒	☐	Dual use research of concern

Methods

n/a	Involved in the study	
☒	☐	ChIP-seq
☐	☒	Flow cytometry
☒	☐	MRI-based neuroimaging

Antibodies

Antibodies used

Cas9 Cell Signaling 97982 AB_2800295
Cytokeratin Agilent M3515 AB_2132885
ISG15 Santa Cruz sc-166755 AB_2126308
phospho-TBK (s172) Cell Signaling 5483 AB_10693472
STING Cell Signaling 13647 AB_2732796
STING R&D Systems MAB7169 AB_10971940
TBK1 Cell Signaling 3504 AB_2255663
α-Tubulin Sigma Aldrich T5618 AB_477579
β-Tubulin Cell Signaling 2128 AB_823664
γH2AX Millipore 05-636 AB_309864
Vinculin Cell Signalong 13901 AB_2728768
Rabbit Anti-Mouse IgG HRP conjugate Millipore AQ160P AB_92795
Goat Anti-Rabbit IgG HRP conjugate Millipore AQ132P AB_92785

Dilutions of all primary antibodies are specified in Supplementary Table 3 as indicated in the methods section of the manuscript.

Validation	Cas9 - Species: Mouse. Validated with western blotting of transfected and control human cell lysates (Cell Signaling and Manuscript)<br>Cytokeratin - Species Mouse. well cited commercially available antibody used for staining cytokeratin in human tissues. Validated by IHC analysis of human head and neck tumor specimens (this manuscript) and skin epithelium (PMID 33257876)<br>ISG15 - Species: mouse. Validation by western blotting in control (human and mouse) lysates with western blotting (SCBT) and this manuscript with STING silencing<br>phospho-TBK1 (s172) - Species Rabbit. Validated with western blotting of human control lysates with phosphatase treatment or LPS stimulation (Cell Signaling)<br>STING Cell Signaling - Species rabbit. Validation by western blotting of control or transfected cell lysates (mouse and human; cell signaling), Control or STING KO cell lysates and Supplemental Figure 3 for full validation in this manuscript<br>STING R&D Systems - Species mouse. Validation by western blotting of Control human cell Lysates (R&D) and in human tumor specimens (this manuscript).<br>TBK1 - Species rabbit. Validation using western blotting of human control and knockout Lysates (Cell Signaling)<br>α-Tubulin - Species mouse. Well published antibody recognizing alpha-tubulin. Immunofluorescent and western blot staining of various cultured cells (human and chicken - Sigma)<br>β-Tubulin - Species Rabbit. Validated by western blotting of human control Lysates (Cell Signaling)<br>γH2AX - Species Mouse. Well published antibody used extensively for the detection of s139 γH2AX. T=Validated by analysis of treated and and untreated human lysates (western blotting) and cells (immunofluorescence; Sigma - Millipore and this manuscript).<br>Vinculin - Species Rabbit. Validated by analysis of various human cell lines (western blotting and IHC - Cell Signaling).

Eukaryotic cell lines

Policy information about cell lines

Cell line source(s)	FaDu and Detroit562 - ATCC<br>MOC1 - Kerafast<br>HEK293T cells used for lentiviral production were a kind gift from Dr. Ryan Jensen (Yale University, New Haven CT).
Authentication	FaDu and Detroit562 cells have been validated by STR profiling (ATCC). No authentication of MOC1 or HEK293T cells was performed.
Mycoplasma contamination	Cell lines tested negative for mycoplasma.
Commonly misidentified lines<br>(See ICLAC register)	No commonly misidentified lines were used

Animals and other organisms

Policy information about studies involving animals; ARRIVE guidelines recommended for reporting animal research

Laboratory animals	4 to 6 week-old female athymic nude mice (Foxn1nu, Envigo; FaDu and Detroit562 xenografts) or C57BL/6J (The Jackson Laboratory; MOC1 xenografts) were used. The mice were housed in accordance to the Yale University Institutional Animal Care and Use Committee (IACUC) guidelines. Yale Animal Resources Center (YARC) ensured that housing temperature was kept at 72 degrees F (+/- 2 degrees), relative humidity 50% (+/-10%), with a 12 hour on/12 hour off light/dark cycle.
Wild animals	No wild animals were used in this study.
Field-collected samples	No field-collected samples were used.
Ethics oversight	All experimental procedures were approved in accordance with IACUC and Yale University institutional guidelines for animal care and ethics and guidelines for the welfare and use of animals in cancer research.

Note that full information on the approval of the study protocol must also be provided in the manuscript.

Human research participants

Policy information about studies involving human research participants

Population characteristics	Please see Supplementary Table 2 for clinical characteristics of YTMA329. Publicly available TCGA data were used as described<br>in methods.
Recruitment	We analyzed retrospectively collected, formalin-fixed, paraffin-embedded (FFPE) tumor specimens which were in TMA format. Specimens were collected and used with specific consent or waiver of consent under the approval from the Yale Human Investigation Committee protocol #9505008219. The HNSCC cohort (YTMA329) contained 186 oropharynx tumors resected between 2001 and 2012 from both primary and metastatic lymph node sites.
Ethics oversight	Yale Human Investigation Committee protocol #9505008219

Note that full information on the approval of the study protocol must also be provided in the manuscript.

Clinical data

Policy information about clinical studies

All manuscripts should comply with the ICMJE guidelines for publication of clinical research and a completed CONSORT checklist must be included with all submissions.

Clinical trial registration	N/A
Study protocol	Specimens were collected and used with specific consent or waiver of consent under the approval from the Yale Human Investigation Committee protocol #9505008219.
Data collection	The HNSCC cohort (YTMA329) contained 186 oropharynx tumors resected between 2001 and 2012 from both primary and metastatic lymph node sites.
Outcomes	Retrospective analysis of PFS.

Flow Cytometry

Plots

Confirm that:

- [x] The axis labels state the marker and fluorochrome used (e.g. CD4-FITC).
- [x] The axis scales are clearly visible. Include numbers along axes only for bottom left plot of group (a 'group' is an analysis of identical markers).
- [x] All plots are contour plots with outliers or pseudocolor plots.
- [x] A numerical value for number of cells or percentage (with statistics) is provided.

Methodology

Sample preparation	For cell cycle cells were treated as indicated and fixed with 70% ethanol and stained with FxCycle PI/RNAse solution per the manufacturers protocol.<br>Cells were plated in 10 cm dishes and irradiated as indicated. Adherent cells were incubated with 5μM CM-H2DCFDA (5-(and-6)-chloromethyl-2′7′-dichlorodihydrofluorescein diacetate acetyl ester; Invitrogen) for 30 min at 37 °C, then the cells were washed once with PBS. Stained cells were collected by trypsinization and resuspended in PBS. ROS generation was assessed by flow cytometry (excitation, 488 nm; emission, 515–545 nm) with 2 × 104 cells for each condition.
Instrument	BD Bioscience LSR II flow cytometer
Software	Data were analyzed with FlowJo software 7.6
Cell population abundance	No cell sorting was performed.
Gating strategy	For cell cycle analysis doublet discrimination was performed using PI-A vs PI-H. FlowJo cell cycle analysis was then performed on samples with these gates applied. For ROS experiments populations were defined by analyzing SSC vs FSC followed by SSCA<br>vs SSC-W to define a population of singlets. The CM-H2DCFDA gate was then set to identify the main cell population.

- [x] Tick this box to confirm that a figure exemplifying the gating strategy is provided in the Supplementary Information.
